# Hyperchloremia and moderate increase in serum chloride are associated with acute kidney injury in severe sepsis and septic shock patients

**DOI:** 10.1186/s13054-016-1499-7

**Published:** 2016-10-06

**Authors:** Bandarn Suetrong, Chawika Pisitsak, John H. Boyd, James A. Russell, Keith R. Walley

**Affiliations:** 1 Centre for Heart Lung Innovation, St. Paul’s Hospital, University of British Columbia, 1081 Burrard Street., Vancouver, BC V6Z 1Y6 Canada; 2 Department of Pediatrics, Faculty of Medicine, Thammasat University, Pathum Thani, Thailand; 3 Department of Anesthesiology, Ramathibodi Hospital, Faculty of Medicine, Mahidol University, Bangkok, Thailand

**Keywords:** Chloride, Hyperchloremia, Acute kidney injury, Sepsis, Septic shock

## Abstract

**Background:**

Acute kidney injury and hyperchloremia are commonly present in critically ill septic patients. Our study goal was to evaluate the association of hyperchloremia and acute kidney injury in severe sepsis and septic shock patients.

**Methods:**

In this retrospective cohort study in a provincial tertiary care hospital, adult patients with severe sepsis or septic shock and serum chloride measurements were included. Serum chloride was measured on a daily basis for 48 hours. Primary outcome was development of acute kidney injury (AKI) and association of AKI and serum chloride parameters was analyzed.

**Results:**

A total of 240 patients were included in the study, 98 patients (40.8 %) had hyperchloremia. The incidence of acute kidney injury (AKI) was significantly higher in the hyperchloremia group (85.7 % vs 47.9 %; *p* < 0.001). Maximal chloride concentration in the first 48 hours ([Cl^-^]_max_) was significantly associated with AKI. In multivariate analysis, [Cl^-^]_max_ was independently associated with AKI [adjusted odds ratio (OR) for AKI = 1.28 (1.02–1.62); *p* = 0.037]. The increase in serum chloride (Δ[Cl^-^] = [Cl^-^]_max_ – initial chloride concentration) demonstrated a dose-dependent relationship with severity of AKI. The mean Δ[Cl^-^] in patients without AKI was 2.1 mmol/L while in the patients with AKI stage 1, 2 and 3 the mean Δ[Cl^-^] was 5.1, 5.9 and 6.7 mmol/L, respectively. A moderate increase in serum chloride (Δ[Cl^-^] ≥ 5 mmol/L) was associated with AKI [OR = 5.70 (3.00–10.82); *p* < 0.001], even in patients without hyperchloremia [OR = 8.25 (3.44–19.78); *p* < 0.001].

**Conclusions:**

Hyperchloremia is common in severe sepsis and septic shock and independently associated with AKI. A moderate increase in serum chloride (Δ[Cl^-^] ≥5 mmol/L) is associated with AKI even in patients without hyperchloremia.

## Background

Acute kidney injury (AKI) frequently occurs in patients with severe sepsis and septic shock [1]. A small rise in serum creatinine (26.5 μmol/L) in critically ill patients is associated with higher mortality, longer length of stay, greater need of vasopressor and mechanical ventilator support and worse long-term outcomes [2–5]. Identifying risk factors for development of AKI in sepsis may therefore be helpful to understand and avoid this profound complication.

Intravenous fluid resuscitation to restore effective circulatory volume in severely ill septic patients is a mainstay of early therapy of severe sepsis and septic shock. The Surviving Sepsis Campaign International Guidelines recommends crystalloids as the initial fluid of choice for resuscitation of septic shock patients [6]. Normal 0.9 % saline solution (Saline), is the most common isotonic crystalloid solution used for resuscitation globally [7–9]. However, the electrolyte composition of Saline is quite different from serum electrolyte composition; Saline has 50 % greater chloride than serum (154 vs 100 mmol/L, respectively) [10]. Consequently, hyperchloremic metabolic acidosis is a common consequence of chloride-rich solution resuscitation [11, 12].

Animal and human studies demonstrate that infusion of Saline results in decreased renal blood flow, reduced glomerular filtration rate, and delayed time to micturition [13–15]. In an animal model of sepsis, resuscitation with Saline resulted in a higher incidence of AKI compared to a balanced crystalloid solution with a more physiologic chloride concentration [16]. A recent study conducted by Yunos and colleagues [17] in critically ill adults found that restriction of chloride-rich fluid was associated with a significant decrease in the incidence of AKI and need for renal replacement therapy (RRT). However, a subsequent randomized controlled trial of normal saline vs. a balanced solution (Plasma-Lyte) found no difference in development of AKI. However, patients were unselected ICU patients, the exposure was only 2 L over the ICU stay, no renal biomarkers were measured, and the serum chloride was not reported. Thus it remains controversial how to manage fluids and chloride in the critically ill. To date, the association of hyperchloremia and AKI has been observed in unselected critically ill patients [18] but not specifically in septic patients [19, 20].

Rapid volume resuscitation of septic patients with Saline rapidly changes serum chloride concentration. Despite all of the above work, it is unclear whether AKI associated with chloride-rich volume resuscitation is due to the absolute value of serum chloride rising above a threshold – hyperchloremia – or whether the change of serum chloride concentration is more important. For example, a rapid increase in serum sodium concentration (rather than the absolute serum sodium concentration) can lead to central pontine myelinolysis.

Accordingly we first tested the hypothesis that hyperchloremia after volume resuscitation was associated with AKI in severe sepsis and septic shock patients. Second, we determined whether the change of serum chloride concentration resulting from volume resuscitation was associated with AKI in severe sepsis and septic shock patients.

## Methods

### Study design and participants

This study was a retrospective cohort study of patients with severe sepsis or septic shock admitted to St. Paul’s Hospital in Vancouver, a tertiary care referral hospital, from January 2011 to April 2015. We included all adults patients with the following criteria: (1) 18 years or older; (2) diagnosis of severe sepsis (defined by two of four Systemic Inflammatory Response Syndrome (SIRS) criteria plus a suspected or confirmed source of infection with at least one organ dysfunction or a lactate greater than 4 mmol/L) or septic shock (defined by sepsis-induced hypotension, tissue hypoperfusion or vasopressor requirement); and (3) initial serum chloride and daily serum chloride concentration for the first 48 hours were measured. We excluded patients with pre-existing chronic renal failure or chronic use of RRT. Written informed consent was obtained from all patients or their representative and the study protocol was approved by our institutional ethics board.

All of these patients were diagnosed via an institutional sepsis protocol in the Emergency Department. Study screening was triggered by this protocol. Most of the resuscitation was conducted by Emergency Department physicians. The primary resuscitation fluid was Saline. Thirty-seven percent of these patients were admitted to the ICU and managed by the attending intensivist. The treatment of these patients was evaluated and directed by attending physicians without a resuscitation guideline or protocol.

### Study variables

Serum chloride concentration was measured by indirect potentiometry (ADVIA 1800 Chemistry System; Siemens Healthcare Diagnostic Inc., Oakville, ON, Canada). The initial chloride concentration, [Cl^-^]_0_ was the initial serum chloride concentration measured at the time that the patient fulfilled diagnostic criteria for severe sepsis or septic shock (above). Serum chloride concentration was measured at least daily for the first 48 hours and the maximal serum chloride concentration during this time period was designated as [Cl^-^]_max_. The increase in serum chloride, Δ[Cl^-^], was the difference between maximal serum chloride level and initial serum chloride level (Δ[Cl^-^] = [Cl^-^]_max_ - [Cl^-^]_0_). Hyperchloremia was defined as [Cl^-^]_max_ ≥ 110 mmol/L [20].

The Acute Physiology and Chronic Health Evaluation II (APACHE II) score was determined by using clinical and laboratory data on the first day of enrollment. AKI was diagnosed and classified by Kidney Disease Improving Global Outcomes (KDIGO) consensus criteria by an increase in serum creatinine concentration > 50 % from a baseline creatinine concentration measured within 3 months prior to enrollment [21]. If a previous serum creatinine was not available, it was calculated by assuming that the glomerular filtration rate (GFR) of the patient was 75 mL/min per 1.73 m^2^ and serum creatinine concentration was computed using the modified 4-variable Modification of Diet in Renal Disease (MDRD) formula [22].

### Study outcomes

The primary outcome was development of AKI by KDIGO criteria. The secondary outcomes were requirement of RRT and 28-day mortality.

### Statistical analysis

Demographic and clinical data were compared between patients with hyperchloremia and without hyperchloremia. Continuous data were reported by using mean ± standard deviation (SD) or median (interquartile range) and categorical data as percentage. We used *t* tests to compare normally distributed continuous data and Wilcoxon signed-rank tests for non-normally distributed data. For categorical variables, a chi-square test was used.

Logistic regression was used to test for association of chloride parameters ([Cl^-^]_0_, [Cl^-^]_max_ and Δ[Cl^-^], hyperchloremia) with AKI, RRT, and 28-day mortality. Univariate logistic regression was used to test for unadjusted association between chloride parameters and AKI. Multivariate logistic regression was used to test for associations between chloride parameters and AKI after adjusting for potentially confounding covariates. Independent variables with *p* < 0.1 in univariate models were incorporated as covariates into the multivariate regression model. These variables consisted of demographic data (age, gender), underlying diseases (hypertension, congestive heart failure, chronic obstructive pulmonary disease) and clinical severity at presentation (APACHE II score, serum lactate, requirement of vasopressor, mechanical ventilator requirement).

All analyses were performed using SPSS, version 20 (SPSS, Chicago, IL, USA). A two-sided *p* value less than 0.05 was considered to be statistically significant.

## Results

### Patient characteristics

**Fig. 1:**
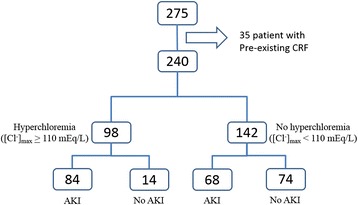
Flow chart of the patients with severe sepsis and septic shock in the study. *[Cl* ^*-*^ *]*_*max*_ maximal chloride concentration in the first 48 hours, *AKI* acute kidney injury, *CRF* chronic renal failure

In this study, 275 patients with severe sepsis and septic shock were enrolled. Thirty-five patients were excluded because they had pre-existing chronic renal failure. Thus, 240 patients were eligible for further evaluation (Fig. 1). Of these, 98 patients (40.8 %) had hyperchloremia within the first 48 hours of resuscitation and 142 patients (59.2 %) did not have hyperchloremia. Demographics, baseline characteristics and clinical outcome of patients with hyperchloremia and without hyperchloremia are shown in Table 1. Patients with hyperchloremia had a higher heart rate and APACHE II score, vasopressor and ventilator requirement. Comorbidities, serum lactate and fluid intake/output were not different between the two groups. Baseline serum creatinine also was not significantly different in both groups. Notably, there was no difference in serum chloride before full resuscitation between those patients who developed hyperchloremia ([Cl^-^]_0_ 104.3 ± 7.7 mmol/L) and those who did not ([Cl^-^]_0_ 103.7 ± 4.6 mmol/L, *p* = 0.51).

**Table 1:** Demographic and clinical variables and outcome of patients classified by serum chloride status

Variable	Hyperchloremia *N* = 98	No hyperchloremia *N* = 142	*p*
Demographics			
Age, yr, mean ± SD	57.5 ± 15.1	52.9 ± 18.4	0.05
Male, %	46.9	55.6	0.38
Underlying diseases			
Hypertension, %	36.7	28.2	0.074
Ischemic heart disease, %	6.1	5.6	0.65
CHF, %	4.1	8.5	0.10
DM, %	21.4	21.1	0.71
COPD, %	21.4	18.3	0.086
Cirrhosis, %	7.1	4.9	0.082
Malignancy, %	4.1	5.6	0.20
HIV, %	11.2	9.2	0.56
Chronic steroid treatment, %	5.1	6.3	0.91
Clinical parameters at presentation			
MAP, mean ± SD	85 ± 21	89 ± 17	0.12
HR, mean ± SD	104 ± 22	96 ± 21	0.003^a^
Lactate, mean ± SD	2.9 ± 2.8	2.5 ± 2.4	0.29
Creatinine(mg/dL), median (IQR)	1.0 (0.7-1.4)	0.9 (0.4-1.4)	0.49
APACHE II score, mean ± SD	11.3 ± 5.0	7.8 ± 5.4	<0.001^a^
Clinical parameters at 24 hr			
Vasopressor, %	31	11	<0.001^a^
Mechanical ventilator, %	64	32	<0.001^a^
Fluid intake, mean ± SD	4959 ± 3,417	4691 ± 2,341	0.71
Urine output, mean ± SD	2416 ± 1,146	2554 ± 1,463	0.644
Chloride parameters			
Initial chloride ([Cl^-^]_0_), mean ± SD	104.3 ± 7.7	103.7 ± 4.6	0.51
Maximal Cl in 48 hours ([Cl^-^]_max_)	104.4 ± 3.7	114.4 ± 3.6	<0.001^a^
Increase in serum Cl (Δ[Cl^-^])	3.2 ± 4.2	6.1 ± 5.0	<0.001^a^
Clinical outcome			
AKI, %	85.7	47.9	<0.001^a^
RRT, %	7.1	3.5	0.206
28-day mortality, %	6.1	1.4	0.066

*SD* standard deviation, *CHF* congestive heart failure, *DM* diabetes mellitus, *COPD* chronic obstructive pulmonary disease, *HIV* human immunodeficiency virus, *MAP* mean arterial pressure, *HR* heart rate, *IQR* interquartile rate, *APACHE* Acute Physiology and Chronic Health Evaluation, *[Cl* ^*-*^ *]*_*0*_ initial chloride concentration, *[Cl* ^-^ *]*_*max*_ maximal chloride concentration in the first 48 hours, *Δ[Cl* ^*-*^ *]* increase in serum chloride; *AKI* acute kidney injury, RRT renal replacement therapy

^a^Indicates statistical significance, *p* < 0.05

### Univariate analysis

#### Serum chloride and acute kidney injury

The initial serum chloride, [Cl^-^]_0_, before full resuscitation was not associated with development of AKI [odds ratio 1.01 (0.97–1.05); *p* = 0.51]. The incidence of AKI was significantly higher in the hyperchloremia group (84 of 98 = 85.7 % versus 68 of 142 = 47.9 %; *p* < 0.001). The maximum chloride concentration within the first 48 hours, [Cl^-^]_max_, was associated with AKI with an odds ratio 1.14 per mmol [Cl^-^] (95 % CI 1.08–1.20, *p* < 0.001) (Table 2). Non-significant trends for patients with hyperchloremia, compared to those without hyperchloremia, included a need of RRT (7.1 % versus 3.5 %; *p* = 0.206) and 28-day mortality (6.1 % versus 1.4 %; *p* = 0.066).

**Table 2:** Univariate logistic regression model to test association of AKI and initial serum chloride ([CL^-^]_0_), maximal serum chloride in first 48 hours ([Cl^–^]_max_), and increase in serum chloride (Δ[Cl^-^] = [Cl^-^]_max_–[CL^-^]_0_)

Variable	AKI stage 1 to 3	*p*	AKI stage 2 and 3	*p*
	Odds ratio (95 % CI)		Odds ratio (95 % CI)	
[CL^-^]_0_	1.01 (0.97–1.05)	0.478	0.99 (0.95–1.03)	0.562
[Cl^-^]_max_	1.14 (1.08–1.20)	<0.001^a^	1.07 (1.02–1.12)	0.006^a^
Δ[CL^-^]	1.25 (1.16–1.36)	<0.001^a^	1.14 (1.07–1.21)	<0.001^a^

*AKI* acute kidney injury, *[Cl* ^*-*^ *]*_*0*_ initial chloride concentration, *[Cl* ^*-*^ *]*_*max*_ maximal chloride concentration in the first 48 hours, *Δ[Cl* ^*-*^ *]* increase in serum chloride, *CI* confidence interval

^a^Indicates statistical significance, *p* <0.05

#### Increase in serum chloride and severity of AKI

**Fig. 2:**
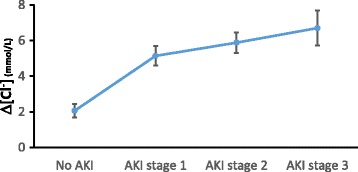
Increase in serum chloride and AKI severity. The mean increase in serum chloride (Δ[Cl^-^]) in AKI stage 1, 2 and 3 is significantly higher than in patients without AKI (*p* < 0.05) and these data suggest a dose-response relationship between Δ[Cl^-^] and AKI stage. *Δ[Cl* ^*-*^ *]* increase in serum chloride, *AKI* acute kidney injury,

The increase in serum chloride, Δ[Cl^-^] (= [Cl^-^]_max_ - [Cl^-^]_0_), was strongly associated with AKI. The odds ratio for development of AKI for Δ[Cl^-^] was 1.25 per mmol Δ[Cl^-^] (95 % CI 1.16–1.36; *p* < 0.001), which was a stronger effect than for [Cl^-^]_max_. Importantly, Δ[Cl^-^] remained significantly associated with development of AKI even in those patients who were never hyperchloremic with an odds ratio of 1.37 per mmol Δ[Cl^-^] (95 % CI 1.20–1.56). A dose-response relationship of Δ[Cl^-^] and severity of AKI was observed; the greater the Δ[Cl^-^], the more severe the AKI stage. The mean Δ[Cl^-^] in patients without AKI was 2.06 mmol/L, for AKI stage 1 Δ[Cl^-^] was 5.14 mmol/L, for AKI stage 2 Δ[Cl^-^] was 5.88 mmol/L, and for AKI stage 3 Δ[Cl^-^] was 6.70 mmol/L (Fig. 2).

#### Moderate increase in serum chloride and AKI

Since AKI stage 1, 2, and 3 were all associated with a mean Δ[Cl^-^] ≥ 5 mmol/L we used this value as a threshold. A moderate increase in serum chloride (Δ[Cl^-^] ≥ 5 mmol/L) identified patients with an odds ratio of developing any AKI of 5.70 (3.00–10.82); *p* < 0.001 and an odds ratio of developing more severe AKI (AKI stage 2 and 3) of 3.40 (1.95–5.94); *p* < 0.001. Interestingly, in patients without hyperchloremia, Δ[Cl^-^] ≥ 5 mmol/L was also associated with AKI (OR = 8.25, 95 % CI 3.44–19.78; *p* < 0.001) and more severe AKI (AKI stage 2 and 3) (OR = 4.8, 95 % CI 2.1–10.7; *p* < 0.001) (Table 3).

**Table 3:** Univariate logistic regression model to test association of AKI and increase in serum chloride (ΔCL = [Cl-]_max_ - CL_0_) in all patients and patients without hyperchloremia

Variable	AKI stage 1 to 3	*p*	AKI stage 2 and 3	*p*
	Odds ratio (95 % CI)		Odds ratio (95 % CI)	
All patients				
Δ[Cl^-^]	1.25 (1.16–1.36)	<0.001^a^	1.14 (1.07–1.20)	<0.001^a^
Δ[Cl^-^] ≥ 5 mmol/l	5.70 (3.00–10.82)	<0.001^a^	3.40 (1.95–5.94)	<0.001^a^
Patients without hyperchloremia				
Δ[Cl^-^]	1.37 (1.20–1.56)	<0.001^a^	1.25 (1.13–1.38)	<0.001^a^
Δ[Cl^-^] ≥ 5 mmol/l	8.25 (3.44–19.78)	<0.001^a^	4.77 (2.13–10.70)	<0.001^a^

*AKI* acute kidney injury, *Δ[Cl* ^*-*^ *]* increase in serum chloride, *[Cl* ^*-*^ *]*_*max*_ maximal chloride concentration in the first 48 hours, *[Cl* ^*-*^ *]*_*0*_ initial chloride concentration, *CI* confidence interval

^a^Indicates statistical significance, *p* < 0.05

#### Multivariate analysis

To adjust for baseline differences we incorporated age, gender and all potentially confounding variables [with *p* < 0.1 in univariate logistic regression analysis; hypertension, congestive heart failure, chronic obstructive pulmonary disease (COPD), APACHE II score, serum lactate, vasopressor and ventilator requirement] into the multivariate model. [Cl^-^]_max_ remained significantly associated with AKI (OR = 1.35, 95 % CI 1.09–1.66, *p* = 0.006). Norepinephrine dosage was included quantitatively in the multivariable model and, while associated with AKI in the univariate analysis, it sufficiently covaried with other factors in the multivariate model (e.g., mean arterial pressure, APACHE II score) so that this association was not significant in the multivariate model. In contrast serum chloride remained an independent predictor of AKI (Table 4).

**Table 4:** Multivariate logistic regression of association of AKI with initial serum chloride ([Cl^-^]_0_), maximal serum chloride ([Cl^-^]_max_), initial serum sodium (Na_0_) and maximal serum sodium(Na_max_)

	OR	95 % confidence interval		*p*
Age	0.99	0.95	1.04	0.776
Sex (male)	0.40	0.09	1.77	0.228
Hypertension	0.68	0.13	3.70	0.656
APACHE II	1.55	1.19	2.03	0.001^a^
Serum lactate	1.60	0.84	3.04	0.155
Norepinephrine dosage	1.06	0.82	1.36	0.680
Ventilator requirement	3.95	0.57	27.44	0.165
[Cl^-^]_0_	0.87	0.70	1.10	0.240
[Cl^-^]_max_	1.28	1.02	1.62	0.037^a^
Δ[Cl^-^]	1.32	1.07	1.61	0.008^a^
Na_0_	1.01	0.76	1.35	0.825
Na_max_	1.03	0.81	1.31	0.825

Multivariate logistic regression model was adjusted by incorporating all potentially confounding factors including age, gender, underlying diseases, initial serum lactate, APACHE II score and requirement of vasopressor and ventilator

*AKI* acute kidney injury, *OR* odds ratio, *APACHE* Acute Physiology and Chronic Health Evaluation

^a^Indicates statistical significance, *p* < 0.05

#### Serum sodium and bicarbonate and AKI

To test an alternative explanation that serum chloride was simply a marker of the degree of volume resuscitation, we repeated the multivariate analysis using alternative electrolytes that would similarly covary with Saline volume resuscitation. The multivariable analysis was repeated using initial and maximal serum sodium and initial and maximal serum bicarbonate. None of these variables were significant independent predictors of AKI (Table 4).

## Discussion

The main result of this study is hyperchloremia is common and significantly associated with AKI in severe sepsis and septic shock patients. Although initial serum chloride concentration before volume resuscitation did not correlate with development of AKI, maximal serum chloride concentration in the first 48 hours ([Cl^-^]_max_) and the increase in serum chloride (Δ[Cl^-^]) were significantly associated with AKI. Because the patients in the hyperchloremia group had higher APACHE II score, vasopressor and ventilator requirement that might result in higher AKI than the patients in the normochloremia group rather than by hyperchloremia, we use a multivariate logistic regression model to minimize the effect of these confounders. After several potential confounders such as age, gender, hypertension, congestive heart failure, COPD, APACHE II score, serum lactate, vasopressor and ventilator requirement were adjusted for in the multivariate logistic regression model, [Cl^-^]_max_ remained independently associated with AKI. Moreover, an increase in serum chloride was associated with AKI and more severe AKI (AKI stage 2 and 3). Importantly, Δ[Cl^-^] ≥ 5 mmol/L was associated with the development of AKI even in the patients who never developed hyperchloremia. Thus, these data suggest that a rapid change in serum chloride concentration may be more important than the absolute value of serum chloride in causing AKI.

To our knowledge this is the first report of the association of a moderate increase in serum chloride (Δ[Cl^-^] ≥ 5 mmol/L) and development of AKI in severe sepsis and septic shock, even in patients without hyperchloremia. Our results are consistent with a retrospective study of unselected critically ill patients in which there was an association of maximal chloride concentration and the development of AKI [18]. Our study used serum chloride concentration in the first 48 hours to define hyperchloremia and found the association of [Cl^-^]_max_ with the development of AKI in severe sepsis and septic shock. We obtained chloride measurements prior to significant volume resuscitation and found that this initial serum chloride concentration ([Cl^-^]_0_) was not associated with development of AKI. Previous studies found a relationship between initial chloride concentration and development of AKI, although these initial measurements may have followed a substantial saline resuscitation [19, 20]. More recently, a double-blind randomized trial evaluating the effect of Saline versus Plasma-Lyte in the intensive care unit (the SPLIT trial) demonstrated no difference of AKI incidence and severity between the two groups [23]. However, most patients were postoperative (71 %) and only 4 % of patients were diagnosed as sepsis. Overall incidence of AKI by KDIGO criteria was lower (27 %) compared to what we observed (63 %) in our sepsis/septic shock population. The volume of crystalloid solution used in that trial (2000 mL) was much less than the average volume that was infused in our patients (4825 mL). This study did not report serum chloride concentrations so it is unknown whether this small volume of Saline resuscitation over an extended period of time was sufficient to cause either hyperchloremia or Δ[Cl^-^] ≥ 5 mmol/L.

We did not find a statistically significant association between hyperchloremia and need for RRT although we observed a trend in this direction. A previous study found a similar statistically significant trend in the association of chloride restrictive strategy and reduction of RRT requirement [17]. However, we excluded the patients with pre-existing chronic renal failure and need of RRT in our study was lower (5 % vs 10 %). Second, a smaller sample size in our study limited the statistical power of detection. Moreover, our populations were exclusively severe sepsis or septic shock while in the previous study only 7 % were severe sepsis or septic shock.

Normal saline solution was originally called “indifferent” saline because it was recognized that human erythrocytes did not lyse in the 0.9 % NaCl solution [24]. Despite being called “normal saline” solution, it has a near physiological concentration of sodium (154 mmol/L) but supraphysiologic concentration of chloride (154 mmol/L or 1.5 times of normal serum chloride concentration) [25]. Infusion of 0.9 % NaCl is significantly associated with hyperchloremic metabolic acidosis in both healthy volunteers and various types of patient [26–35]. The clinical significance of iatrogenic hyperchloremic metabolic acidosis remains uncertain. The hospital mortality is much higher in lactic acidosis than in hyperchloremic acidosis (56 % vs 29 %) compared with the hospital mortality of patients without acidosis (26 %) [36].

Supraphysiologic serum chloride concentration has detrimental effects on renal function. In animal models, infusion of 0.9 % NaCl resulted in renal vasoconstriction and decreased renal blood flow and glomerular filtration rate [13, 37–39] possibly due to chloride-induced thromboxane release [38] and augmented response to renal vasoconstrictors such as angiotensin II [40]. Furthermore, the tubuloglomerular feedback mechanism initiated by detection of chloride at the macula densa results in afferent arteriolar vasoconstriction, mesangial contraction and decreased glomerular filtration rate [41]. In healthy human volunteers, infusion of normal saline caused reductions in renal artery blood flow, renal cortical perfusion [14] and delayed first micturition [15, 27]. Saline infusion resulted in a higher incidence of AKI when compared with balanced crystalloids [17, 42, 43]. In an animal model of sepsis, resuscitation of septic animals with Saline resulted in not only higher incidence of AKI and greater severity of AKI but also higher inflammatory mediator concentrations (IL-6) when compared to Plasma-Lyte [16]. Interestingly, the study of effect of an acute Saline infusion on fluid and electrolyte metabolism revealed that the human body required 2 days to restore electrolyte and fluid balance to equilibrium [44].

There are several limitations to this study. First, it was a retrospective cohort study so the association of hyperchloremia with AKI does not necessarily imply causality. Second, we were not able to determine the amount of chloride administered from the medical records so the analysis is based on the consequence – the change in serum chloride concentration. Hyperchloremia may have negative effects on renal function and cause AKI. Alternatively, increased severity of shock would lead to AKI and increased administration of Saline without a causal relationship between the two. Second, these results do not address the mechanism or pathophysiology of hyperchloremia and AKI. Finally, the small sample size significantly limits power to detect an association of hyperchloremia or Δ[Cl^-^] with RRT and 28-day mortality.

## Conclusions

Hyperchloremia is common in severe sepsis and septic shock patients and is independently associated with AKI. Initial serum chloride concentration is not associated with the development of AKI while hyperchloremia, defined as the maximum serum chloride concentrations measurement in the first 48 hours, is associated with development of AKI. Our most striking finding is the association of moderate increase in serum chloride (Δ[Cl^-^] ≥ 5 mmol/L) and AKI even in patients who had serum chloride within the normal range. Finally, there is a continuing need for RCTs of intravenous fluid in sepsis and septic shock that address the risks of saline vs. balanced solutions for development of AKI. Perhaps serum chloride could be a biomarker for response to various intravenous fluids.

## Abbreviations

[Cl^-^]_0_: initial chloride concentration
[Cl^-^]_max_: maximal chloride concentration in the first 48 hours
Δ[Cl^-^]: increase in serum chloride
AKI: acute kidney injury
APACHE: Acute Physiology and Chronic Health Evaluation
CI: confidence interval
COPD: chronic obstructive pulmonary disease
CRF: chronic renal failure
GFR: glomerular filtration rate
KDIGO: Kidney Disease Improving Global Outcomes
MDRD: Modification of Diet in Renal Disease
Na_0_: initial serum sodium
Na_max_: maximal serum sodium in first 48 hours
OR: odds ratio
RRT: renal replacement therapy
SD: standard deviation
SIRS: systemic inflammatory response syndrome
